# Fosaprepitant Weekly vs Every 3 Weeks for the Prevention of Concurrent Chemoradiotherapy–Induced Nausea and Vomiting: A Pilot Randomized Clinical Trial

**DOI:** 10.1001/jamanetworkopen.2023.26127

**Published:** 2023-07-27

**Authors:** Qi Yang, Xiong Zou, Yu-Long Xie, Chao Lin, Yan-Feng Ouyang, Yong-Long Liu, Chong-Yang Duan, Rui You, You-Ping Liu, Rong-Zeng Liu, Pei-Yu Huang, Ling Guo, Yi-Jun Hua, Ming-Yuan Chen

**Affiliations:** 1 Department of Nasopharyngeal Carcinoma, Sun Yat-sen University Cancer Center, State Key Laboratory of Oncology in South China, Collaborative Innovation Center for Cancer Medicine, Guangdong Key Laboratory of Nasopharyngeal Carcinoma Diagnosis and Therapy, Guangzhou, China; 2 Department of Biostatistics, School of Public Health, Southern Medical University, Guangzhou, China

## Abstract

**Importance:**

Unlike substantial evidence in the prevention of chemotherapy-induced nausea and vomiting (CINV), research in the prevention of nausea and vomiting caused by concurrent chemoradiotherapy (CCRT) is currently lacking.

**Objective:**

To compare the efficacy and safety of fosaprepitant weekly vs every 3 weeks for the prevention of nausea and emesis caused by CCRT among patients with nasopharyngeal carcinoma.

**Design, Setting, and Participants:**

This pilot randomized clinical trial was conducted at a single cancer center from November 24, 2020, to July 26, 2021, among patients with nasopharyngeal carcinoma who had achieved CINV control after 2 to 3 cycles of induction chemotherapy. Efficacy analyses were performed in the intention-to-treat population. Data were analyzed on November 4, 2022.

**Interventions:**

Eligible patients were randomly assigned (1:1) to receive fosaprepitant either weekly or every 3 weeks.

**Main Outcomes and Measures:**

The primary end point was the proportion of patients with sustained complete response (defined as no emesis and no rescue therapy) during CCRT. Secondary end points were sustained no emesis, no nausea, no significant nausea, mean time to first emetic episode, quality of life, and 1-year progression-free survival (PFS).

**Results:**

A total of 100 patients (mean [SD] age, 46.6 [10.9] years; 83 [83.0%] male) who had achieved CINV control after induction chemotherapy were randomly assigned to receive fosaprepitant weekly (50 patients) or every 3 weeks (50 patients). There was no significantly significant difference in cumulative risk of emesis or rescue therapy in the group that received weekly fosaprepitant compared with those who received fosaprepitant every 3 weeks (subhazard ratio, 0.66 [95% CI, 0.43-1.02]; *P* = .06). The proportion of patients with sustained no emesis (38% vs 14%; *P* = .003) or no significant nausea (92% vs 72%; *P* = .002) was significantly higher in the group that received fosaprepitant weekly vs those who received fosaprepitant every 3 weeks. Treatments were well tolerated. Patients in the weekly group had improved scores for multiple quality-of-life measures. There was no significant difference in survival outcomes between groups (91.8% vs 93.7%; *P* = .99). In the mean brainstem dose subgroups, a possible treatment interaction effect was observed in sustained complete response (mean brainstem dose ≥36 Gy: hazard ratio [HR], 0.32 [95% CI, 0.15-0.69]; mean brainstem dose <36 Gy: HR, 0.95 [95% CI, 0.55-1.63]) and sustained no emesis (mean brainstem dose ≥36 Gy: HR, 0.21 [95% CI, 0.08-0.53]; mean brainstem dose <36 Gy: HR, 0.73 [95% CI, 0.41-1.28]).

**Conclusions and Relevance:**

In this pilot randomized clinical trial, there was no statistically significant difference in the complete response primary end point, but patients receiving weekly fosaprepitant were less likely to experience emesis compared with those who received fosaprepitant every 3 weeks, especially in the subgroup with a mean brainstem dose of 36 Gy or more. Weekly fosaprepitant was well tolerated and improved quality of life of patients without compromising survival.

**Trial Registration:**

ClinicalTrials.gov Identifier: NCT04636632

## Key Points

**Question:**

Is intensified weekly fosaprepitant more effective than fosaprepitant every 3 weeks in providing sustained protection against nausea and emesis caused by concurrent chemoradiotherapy?

**Findings:**

In this randomized clinical trial of 100 patients with nasopharyngeal carcinoma, there was no significantly significant difference in cumulative risk of emesis or rescue therapy in patients receiving weekly fosaprepitant compared with those receiving fosaprepitant every 3 weeks. Patients who received fosaprepitant weekly were less likely to experience emesis and nausea, a secondary outcome of the trial.

**Meaning:**

This randomized clinical trial found no difference between weekly and thrice-weekly fosaprepitant for the primary outcome of complete response.

## Teaser

This pilot randomized clinical trial assesses the safety and efficacy of fosaprepitant weekly vs every 3 weeks for the prevention of nausea and vomiting caused by concurrent chemoradiotherapy among patients with nasopharyngeal carcinoma.

## Introduction

Nausea and vomiting are still perceived as major problems that result in substantial impairments in the patient’s functional activity and quality of life in patients receiving radiotherapy alone or with concomitant chemotherapy.^1,2,3^ Nausea and vomiting can also lengthen the overall radiotherapy time; sometimes patients even refuse further treatment, which can have serious influences on the treatment adherence and survival outcome.^4,5,6^ Unlike substantial evidence in the prevention of chemotherapy-induced nausea and vomiting (CINV), research in the prevention of nausea and vomiting caused by concurrent chemoradiotherapy (CCRT) is currently lacking. The GAND-emesis study has investigated a neurokinin-1 receptor antagonist (NK-1RA) during radiotherapy and concomitant weekly cisplatin in cervical cancer,^1^ but the emetic risk of concomitant low-dose cisplatin (40 mg/m^2^) is less than high-dose cisplatin. To our knowledge, there has been no evidence regarding the best antiemetic prophylaxis during combined radiotherapy–highly emetogenic chemotherapy (HEC).

Current guidelines suggest that patients undergoing radiotherapy and concomitant chemotherapy should receive antiemetic prophylaxis according to the guidelines for chemotherapy if the risk level of concomitant chemotherapy is higher than that of radiotherapy.^7^ As for the best antiemetic prophylaxis in nasopharyngeal carcinoma (NPC) during CCRT, a combination therapy with a NK-1RA plus a 5-hydroxytryptamine-3 receptor antagonist (5-HT3RA) and a corticosteroid for the prevention of CINV-induced by concomitant HEC should be recommended as the standard treatment, especially considering that radiation of the head and neck has traditionally been regarded as a low–emetic risk treatment.^8^ However, the current guideline recommendations mainly come from the pre–intensity-modulated radiation therapy (IMRT) era.^4,7,8^ Recently, a higher incidence of nausea and vomiting has been reported in patients with head and neck squamous cell carcinomas (HNSCC) treated with IMRT due to a higher dose deposited on the brainstem, considering the conformity,^9,10,11,12,13,14^ and nausea and vomiting in NPC during IMRT may be underestimated.^4,15^ Therefore, a need exists for an intensified antiemetic prophylaxis to improve control of nausea and emesis during combined radiotherapy-HEC.

Fosaprepitant is the prodrug of the NK-1RA aprepitant and can be rapidly converted to the active form after intravenous (IV) administration.^16,17,18^ It has been established that NK-1RA played the antiemetic role at the level of the brainstem, which might be the same site involved in IMRT-induced nausea and vomiting in HNSCC.^19,20,21,22,23,24^ Additionally, the availability of the IV route is more feasible in patients with oral mucositis, which is the most common radiation-induced toxic effect during CCRT.^25,26,27,28^ Our pilot randomized clinical trial study was undertaken to compare the efficacy and safety of fosaprepitant weekly vs every 3 weeks for the prevention of nausea and emesis caused by combined radiotherapy-HEC among patients with NPC.

## Methods

### Study Design

This open-label, prospective, pilot randomized clinical trial was approved by the ethical committee of Sun Yat-Sen University Cancer Center. This study was investigator initiated and conducted at a single institution. Written informed consent was obtained from all patients. This study followed the Consolidated Standards of Reporting Trials (CONSORT) reporting guideline. The trial protocol and statistical analysis plan are provided in Supplement 1.

The inclusion criteria were previously untreated, histologically confirmed nonkeratinizing NPC; stage II to IVa disease (according to the American Joint Committee on Cancer classification system, eighth edition); achieved CINV control (defined as no emesis and no rescue therapy during the first 120 hours after initiation of chemotherapy) using fosaprepitant-based regimens after 2 to 3 cycles of induction chemotherapy; scheduled to receive IMRT and concomitant cisplatin every 3 weeks at 100 mg/m^2^; and adequate hematological function (leucocyte count ≥4000/μL [to convert to ×10^9^/L, multiply by 0.001], hemoglobin ≥9.00 g/dL [to convert to grams per liter, multiply by 10], and platelet count ≥100 × 10^3^/μL [to convert to ×10^9^/L, multiply by 1]), hepatic function (alanine aminotransferase and aspartate aminotransferase ≤2.0 times the upper limit of the reference ranges), and renal function (creatinine clearance ≥60 mL/min/1.73 m^2^ [to convert to millimeters per second per meter-squared, multiply by 0.0167]). The major exclusion criteria were inability to read, comprehend, and finish questionnaires; history of prior malignant neoplasm; history of central nervous system disease (eg, a seizure disorder or brain metastases); administered drugs with antiemetic activity within the 24 hours before receiving the first dose of study medication; and hypersensitivity history of fosaprepitant, 5-HT3RAs, or dexamethasone.

Eligible patients were enrolled after induction chemotherapy. Patients were then randomized 1:1 to receive either fosaprepitant weekly or every 3 weeks during CCRT, with a block size of 6 (known only to the data management team). Random assignment was performed by sealed envelopes at the Clinical Trials Centre of Sun Yat-sen University Cancer Centre, with a computer-generated random number code. Treatment group assignment was not masked.

### Regimens and Assessment

Patients received IMRT and concomitant cisplatin every 3 weeks at 100 mg/m^2^ for 2 cycles. Randomization was conducted 1 day before the first cycle of concomitant cisplatin. All patients received a 5-HT3RA (0.25 mg of palonosetron IV, 1 mg of granisetron IV, or 8 mg of ondansetron IV, with the specific agent chosen by the primary clinician) on day 1 and 10 mg of dexamethasone IV on days 1 through 4 of concomitant cisplatin every 3 weeks. In addition, 150 mg of fosaprepitant was given weekly or every 3 weeks as a 20- to 30-minute IV infusion from the first dose of cisplatin. Patients were allowed to receive rescue therapy, which was defined as any medication prescribed by the clinician to relieve nausea and vomiting.

Patients completed a study diary throughout the study to record the degree of nausea, the timing and number of emetic episodes, and the use of rescue therapy. Patients estimated the extent of their nausea using a validated 4-graded scale (none, mild, moderate, or severe).^29^ If emesis occurred or rescue therapy was used, whichever occurred first, the patient was censored from the study thereafter. Patients were also asked weekly during treatment to complete 2 self-administered questionnaires, the European Organization for Research and Treatment of Cancer (EORTC) Quality-of -Life Questionnaire C30 (QLQ C30) and the EORTC QLQ Head and Neck Cancer–Specific Module (H&N35)^30^ to assess the effect of nausea and emesis on their quality of life.

The primary end point was the proportion of patients with sustained complete response (defined as no emesis and no rescue therapy) during CCRT. Secondary end points were the proportion of patients with sustained no emesis, sustained no nausea, sustained no significant nausea (defined as no or mild nausea), and the mean time to first emetic episode during CCRT. Other outcomes included the quality of life and 1-year progression-free survival (PFS), defined as the time from random assignment to locoregional relapse or distant metastasis, or death from any cause.

### Statistical Analysis

This study followed a selection (or *pick-the-winner*) design,^31^ which compares 2 possible groups for a larger, phase III trial. Prior to the study, our previous study showed a no-emesis rate of 45.4% during CCRT.^32^ In this study, the primary end point was sustained complete response rate, including no emesis and no rescue therapy, which is likely to be lower than 45.4%. We thus estimated a sustained complete response rate of 40%. Based on this value and assuming a 20% difference between groups, a sample size of 36 patients (18 per group) would provide an 85.6% probability of correctly selecting the superior group. A total sample size of 40 was planned to allow for an approximately 10% dropout rate.

Efficacy analyses were performed in the intention-to-treat population, which included all randomly assigned patients. For the cumulative incidence of emesis and other efficacy end points, Gray test was performed to compare groups. Patients were treated as competing events at the time they dropped out from the study for reasons other than emesis. Quality of life was analyzed using a mixed-effects model with EORTC-QLQ C30 and QLQ H&N35 scores assessed each week during treatment as a response value, treatment group and visit time as fixed effects, baseline scores as a covariate, and each patient as a random effect. Categorical variables were compared using the χ^2^ test or Fisher exact test, as indicated. PFS was estimated using Kaplan-Meier method, and the differences were compared by log-rank test. A post-hoc exploratory analysis for covariate interaction of the treatment effect was done to explore whether the treatment effect of fosaprepitant weekly vs every 3 weeks differs among specific covariate groups. Covariates included patient factors (ie, sex and age), tumor factors (ie, T category, N category, and clinical stage), dosimetric factors associated with nausea and vomiting in IMRT, and antiemetic prophylaxis intervention (ie, treatment group). Two previously reported dosimetric factors associated with nausea and vomiting in IMRT were used: mean dose of 36 Gy or more for brainstem^33^ and V40 (percentage volume receiving ≥40 Gy) more than 80% for total vestibule.

All statistical tests were 2-sided, and *P* < .05 was deemed statistically significant. Analyses were performed using SPSS version 20 (IBM) and R version 4.1.2 (R Project for Statistical Computing). Data were analyzed on November 4, 2022.

## Results

### Baseline Characteristics

Between November 24, 2020, and July 26, 2021, 211 patients were screened for eligibility. After the exclusion of 111 patients for ineligibility, the remaining 100 patients (mean [SD] age, 46.6 [10.9] years; 83 [83.0%] male) were randomly assigned to receive fosaprepitant weekly (50 patients) or every 3 weeks (50 patients) (eFigure 1 in Supplement 2). The most common reason for exclusion was that the patients did not achieve CINV control after all cycles of cisplatin-based induction chemotherapy (71 patients). Baseline characteristics and dosimetric parameters were well balanced between groups (eTable 1 and eTable 2 in Supplement 2).

Most patients (97 patients [97.0%]) completed all the treatment cycles, and only 3 patients discontinued (for reasons other than emesis) because of cancellation of cisplatin treatment due to a creatinine clearance of less than 60 mL/min/1.73 m^2^ (1 patient), a hemoglobin level less than 6.0 g/dL (1 patient) and a platelet count less than 100 × 10^3^/μL (1 patient).

### Efficacy

**Figure 1.**
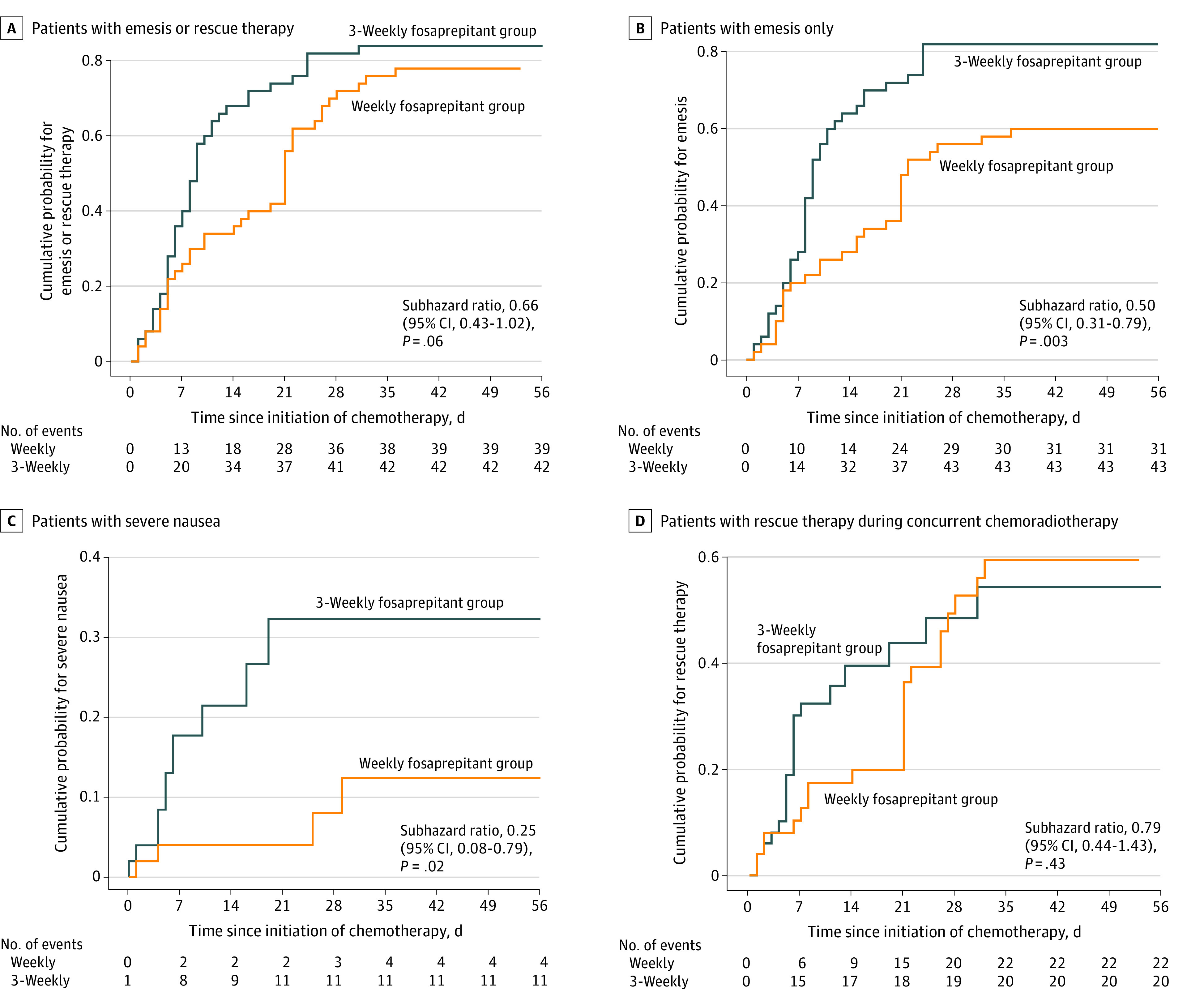
Proportion of Patients With Nausea, Emesis, or Rescue Therapy

The proportion of patients with sustained complete response during CCRT was 20.4% (95% CI, 10.7%-34.8%) for the group receiving weekly fosaprepitant compared with 12.5% (95% CI, 5.1%-25.9%) for those receiving fosaprepitant every 3 weeks. There was no statistically significant difference in cumulative risk of emesis or rescue therapy between groups (subhazard ratio, 0.66 [95% CI, 0.43-1.02]; *P* = .06) (Figure 1).

The proportion of patients with no emesis in the group receiving weekly fosaprepitant was significantly higher than that in patients receiving fosaprepitant every 3 weeks in days 1 to 21 (26 patients [52.0%] vs 14 patients [28.0%]; *P* = .005) and in the overall period (days 1-56: 29 patients [58.0%] vs 7 patients [14.0%]; *P* = .003) (Table 1). Likewise, there were significant differences between groups with respect to freedom from clinically significant nausea (ie, no or mild nausea with no or only little interference with daily activities, as defined in previous trials).^1,29,34^ A total of 49 patients (98.0%) receiving weekly fosaprepitant had no clinically significant nausea, compared with 39 patients (78.0%) receiving fosaprepitant every 3 weeks in days 1 to 21 (*P* = .001); results were similar during the overall period (46 patients [92.0%] vs 36 patients [72.0%]; *P* = .002). During the observation period, none of the patients were continuously free from nausea. No significant differences were observed in the use of rescue therapy in the days 1 to 21 and overall periods. The percentage of patients with no rescue therapy in the weekly fosaprepitant group was significantly higher than that in the fosaprepitant every 3 weeks group, but this was only noted on days 1 to 7. The mean (SD) time to the first emetic episode was 26.5 (16.9) days in the weekly fosaprepitant group and 15.6 (14.3) days in the fosaprepitant every 3 weeks group.

**Table 1.** Efficacy of Fosaprepitant Received Weekly vs Every 3 Weeks

Outcome	Patients, No. (%)^a^		*P* value^b^
	Weekly group (n = 50)	Triweekly group (n = 50)	
Complete response			
Day 1	48 (96)	47 (94)	.65
Days 1-7	37 (74)	29 (58)	.16
Days 1-21	22 (44)	13 (26)	.02
Days 1-56^c^	10 (20)	6 (12)	.06
No emesis			
Day 1	49 (98)	48 (96)	.57
Days 1-7	40 (80)	36 (72)	.35
Days 1-21	26 (52)	14 (28)	.005
Days 1-56^c^	19 (38)	7 (14)	.003
No use of rescue medication			
Day 1	48 (96)	48 (96)	>.99
Days 1-7	44 (88)	35 (70)	.04
Days 1-21	35 (70)	32 (64)	.17
Days 1-56^c^	27 (54)	28 (56)	.43
No significant nausea			
Day 1	49 (98)	46 (92)	.56
Days 1-7	48 (96)	43 (86)	.07
Days 1-21	48 (96)	40 (80)	.02
Days 1-56^c^	45 (90)	37 (74)	.02

^a^ Percentages are point estimates of the final day in the respective assessment periods.

^b^ Calculated using Gray test.

^c^ Time after the first administration of cisplatin.

### Safety

Weekly fosaprepitant was well tolerated. There were no significant differences between the weekly and every 3 weeks groups in the proportions of patients who had at least 1 adverse event (grade ≥1) or who had at least 1 serious adverse event (grade ≥3) (Table 2). None of the serious adverse events were attributed to fosaprepitant by the attending clinician. No grade 4 or 5 adverse events were reported in the study. The incidence of grade 3 adverse events was generally low, and there was no significant difference between groups.

**Table 2.** Adverse Events for Treatment With Fosaprepitant Weekly vs Every 3 Weeks^a^

Event	Grade ≥1			Grade ≥3		
	Patients, No. (%)		*P* value	Patients, No. (%)		*P* value
	Weekly group (n = 50)	Triweekly group (n = 50)		Weekly group (n = 50)	Triweekly group (n = 50)	
Nonhematologic						
Constipation	5 (10)	10 (20)	.16	0	0	NA
Hiccups	2 (4)	2 (4)	>.99	0	0	NA
Dizziness	3 (6)	8 (16)	.11	0	0	NA
Dry mouth	47 (94)	46 (92)	.70	0	0	NA
ALT increase	5 (10)	3 (6)	.71	0	0	NA
AST increase	2 (4)	4 (8)	.67	0	0	NA
Creatinine increase	12 (24)	6 (12)	.12	0	0	NA
Total bilirubin	2 (4)	2 (4)	>.99	0	0	NA
Hematologic						
Leukopenia	5 (10)	2 (4)	.43	3 (6)	0	.24
Neutropenia	2 (4)	3 (6)	>.99	0	0	NA
Anemia	35 (70)	35 (70)	>.99	1 (2)	1 (2)	>.99
Thrombocytopenia	0	2 (4)	.48	0	1 (2)	>.99

Abbreviations: ALT, alanine aminotransferase; AST, aspartate aminotransferase; NA, not applicable.

^a^ No grade 4 or 5 adverse events occurred during treatment.

Adherence rates of the quality-of-life questionnaires deteriorated during the CCRT, with 98% of the expected forms received at baseline, 90% at week 1, 87% at week 2, 88% at week 3, 82% at week 4, 80% at week 5, 73% at week 6, 26% at week 7, and 5% at week 8. Quality-of-life analyses showed that patients who received weekly fosaprepitant experienced less fatigue, nausea and vomiting, dyspnea, insomnia, and appetite loss and presented with better role function and emotional function compared with those who received fosaprepitant every 3 weeks. As for the symptoms and functions of the head and neck, patients receiving weekly fosaprepitant were found to have significantly less problems regarding pain, swallowing, sense, teeth, xerostomia, sticky saliva, coughing, feeling ill, and nutritional supplements (Table 3; eFigure 2 in Supplement 2).

**Table 3.** Mean Differences in Quality-of-Life Scores Between Treatment Groups

Item	Mean difference (95% CI)^a^	*P* value
QLQ-C30		
Physical functioning	−0.92 (−3.77 to 1.93)	.53
Role functioning	5.74 (2.06 to 9.43)	.002
Emotional functioning	3.59 (0.53 to 6.66)	.02
Cognitive functioning	1.83 (−1.14 to 4.81)	.23
Social functioning	6.23 (3.02 to 9.45)	<.001
Global health status	1.68 (−0.78 to 4.14)	.18
Fatigue	−12.06 (−15.03 to −9.10)	<.001
Nausea and vomiting	−10.52 (−13.46 to −7.58)	<.001
Pain	−0.23 (−3.39 to 2.94)	.89
Dyspnea	−8.71 (−11.56 to −5.86)	<.001
Insomnia	−4.40 (−7.64 to −1.15)	.008
Appetite loss	−15.02 (−18.65 to −11.40)	<.001
Constipation	0.22 (−2.84 to 3.29)	.89
Diarrhea	0.87 (−2.05 to 0.32)	.15
Financial difficulties	−1.02 (−4.97 to 2.93)	.61
QLQ-H&N35		
Pain	−3.47(−5.90 to −1.04)	.005
Swallowing	−4.48 (−7.41 to −1.55)	.003
Sense problems	−4.09 (−7.22 to −0.96)	.01
Speech problems	−0.46 (−2.69 to 1.77)	.68
Social eating	−2.25 (−5.09 to 0.59)	.12
Social contact	0.77 (−1.88 to 3.41)	.57
Sexuality	−1.36 (−6.24 to 3.52)	.58
Teeth	−3.69 (−6.14 to −1.23)	.003
Opening mouth	−0.80 (−3.19 to 1.59)	.51
Dry mouth	−8.25 (−11.88 to −4.63)	<.001
Sticky saliva	−9.74 (−13.63 to −5.85)	<.001
Coughing	−4.21 (−7.17 to −1.25)	.005
Felt ill	−10.65 (−14.55 to −6.75)	<.001
Pain killers	−3.84 (−9.35 to 1.68)	.17
Nutritional supplements	−17.34 (−24.12 to −10.57)	<.001
Feeding tube	−0.73 (−3.97 to 2.52)	.66
Weight loss	−4.30 (−11.27 to 2.66)	.23
Weight gain	0.21 (−2.58 to 3.00)	.88

Abbreviations: QLQ-C30, Quality of Life Questionnaire C30; QLQ-H&N35, Quality of Life Questionnaire Head and Neck–35.

^a^ Calculated as weekly group score − 3-weekly group score.

For all 100 patients, the median (IQR) follow-up time was 15.1 (13.8-17.3) months. As of November 4, 2022, 5 progression events occurred in the weekly fosaprepitant group and 5 occurred in the fosaprepitant every 3 weeks group. The patterns of failure are summarized in eTable 3 in Supplement 2. No deaths were recorded. There was no significant difference in survival outcomes between groups. The 1-year PFS and was 91.8% (95% CI, 84.2%-99.4%) in the weekly fosaprepitant group and 93.7% (95% CI, 86.6%-100.0%) in the fosaprepitant every 3 weeks group (*P* = .99) (eFigure 3 in Supplement 2).

### Exploratory Analysis

**Figure 2.**
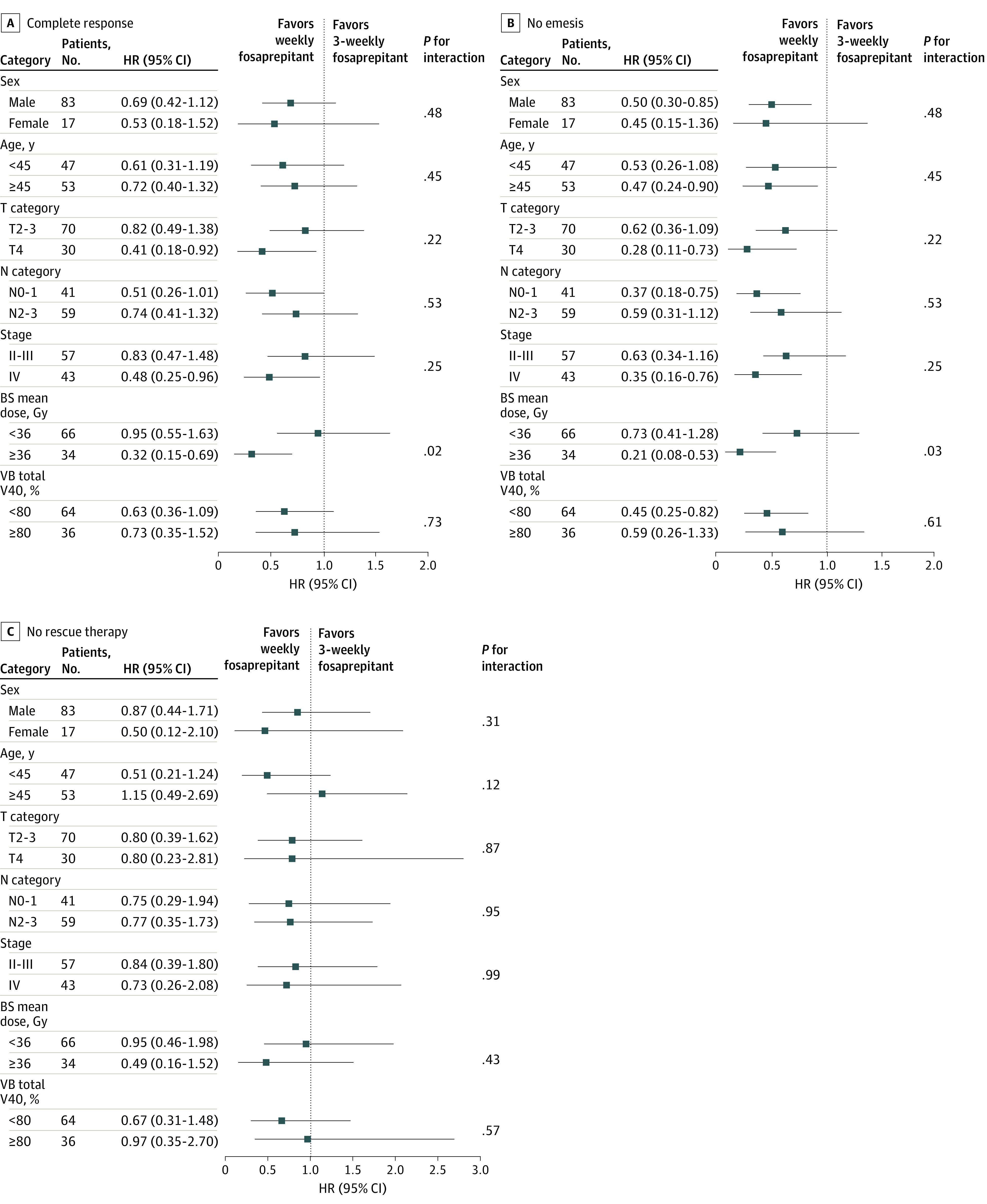
Forest Plots for Complete Response, No Emesis, and No Rescue Therapy BS indicates brainstem; HR, hazard ratio; V40, percentage volume receiving ≥40 Gy; VB, vestibule.

We performed a post-hoc exploratory analysis to investigate whether the effect of the experimental treatment varied for the specific subgroups (Figure 2). The interaction analyses revealed a possible treatment interaction effect in the mean brainstem dose subgroups on sustained complete response (mean brainstem dose ≥36 Gy: hazard ratio [HR], 0.32 [95% CI, 0.15-0.69]; mean brainstem dose <36 Gy: HR, 0.95 [95% CI, 0.55-1.63]) and sustained no emesis (mean brainstem dose ≥36 Gy: HR, 0.21 [95% CI, 0.08-0.53]; mean brainstem dose <36 Gy: HR, 0.73 [95% CI, 0.41-1.28]).

## Discussion

To our knowledge, this randomized clinical trial is the first study to assess the efficacy and safety of fosaprepitant during combined radiotherapy-HEC. Although there was no statistically significant difference in the complete response primary end point, we found that weekly fosaprepitant was more effective in providing sustained protection against emesis and significant nausea than fosaprepitant every 3 weeks. The interactions between treatment effects and mean brainstem dose of 36 Gy or more indicated the benefit of intensified weekly fosaprepitant was more evident in the subgroup with a mean brainstem dose of at least 36 Gy. The tolerability profile, quality-of-life data, and survival outcomes showed that weekly fosaprepitant was well tolerated and improved quality of life of patients without compromising survival.

To our knowledge, there has been no evidence for the best antiemetic prophylaxis during combined radiotherapy-HEC. Several studies established a 3-drug combination regimen to prevent the nausea and vomiting in patients receiving HEC or moderately emetogenic chemotherapy during the first 120 hours after initiation of chemotherapy.^35,36,37,38,39^ The GAND-emesis study extended the 3-drug regimen to the prevention of nausea and vomiting during combined radiotherapy–moderately emetogenic chemotherapy (cisplatin 40 mg/m^2^) in women with cervical cancer.^1^ But the question of whether patients need a more intensified antiemetic prophylaxis during combined radiotherapy-HEC is still open.

In this study, the weekly fosaprepitant regimen provided no significant improvements in sustained no emesis and no significant nausea during CCRT, except before day 1 and days 1 to 7 postcisplatin in cycle 1. This finding suggests that the weekly administration of fosaprepitant was effective for the prevention of emesis and significant nausea after days 1 through 7 and during CCRT. Furthermore, there is now a consensus that the prevention of CINV should be assessed during the acute (day 1), delayed (days 2-5), and overall (days 1-5) phases, especially when considering that the time to first emesis rarely exceeds 5 days. In this study, the mean time to first emesis was 26.5 days in the weekly fosaprepitant group and 15.6 days in the fosaprepitant every 3 weeks group. These results highlight the value of weekly administration of fosaprepitant during combined radiotherapy-HEC due to the increased risk of emesis beyond the first 7 days of the first cycle of cisplatin.

Currently, prevention and control of nausea are more challenging than prevention and control of vomiting.^40,41^ In this study, none of the patients in either group were continuously free from nausea. However, significantly more patients receiving weekly fosaprepitant had no significant nausea across the overall observation period. This modest effect of NK-1RA on nausea is in line with results from previous studies of CINV.^37,38,42,43^

The tolerability profiles in both groups were similar and were mostly associated with the chemotherapeutic agents. We observed a significant improvement in most of the quality-of-life scales in the weekly group owing to the reduction in gastrointestinal toxic effects. Moreover, the adherence rate of weekly fosaprepitant was satisfactory, probably because the IV administration of fosaprepitant provided greater treatment flexibility and convenience for patients with oral mucositis who could not tolerate oral medications.^25,26,27,28^

A possible treatment interaction effect on sustained complete response and sustained no emesis in the mean brainstem dose subgroups was observed. The benefit of weekly fosaprepitant was more evident in patients with a mean brainstem dose of 36 Gy or greater. It is interesting to note that areas connected to the development of emesis also exist in the brainstem. Multiple studies have reported a radiation dose–response association of brainstem dose with nausea and vomiting symptoms, which may contribute to a higher incidence of nausea and vomiting in patients with HNSCC treated with IMRT; these results suggest that radiation dose to the brainstem outside of the target volume influences the incidence of vomiting during IMRT for HNSCC, including NPC.^9,10,11,12,13^ At the same time, several lines of evidence have indicated that NK-1RA may play the antiemetic role at the level of the brainstem.^19,20,21,22,23,24^ As a result, intensified weekly NK-1RA fosaprepitant may have more remarkable antiemetic action in patients with higher mean brainstem dose.

### Limitations

This study has some limitations. Moving forward, it is critical to validate these results with a larger multicenter randomized clinical trial. Second, a computer-based centralized random assignment instead of sealed envelopes would allow for a more accurate allocation procedure by preventing premature revealing. Third, patients who experienced CINV during the induction phase could have been included in the analysis when evaluating determinants of sustained complete control. In addition, there is need for improvement in control of nausea both prophylactically and as rescue therapy during combined radiotherapy-HEC. Antiemetic regimens, including olanzapine, in chemoradiotherapy should be investigated in future clinical trials, because olanzapine is considered to be particularly effective against nausea.

## Conclusions

This randomized clinical trial found that although there was no statistically significant difference in the complete response primary outcome, patients receiving weekly fosaprepitant were less likely to experience emesis compared with those receiving fosaprepitant every 3 weeks, especially in the subgroup with a mean brainstem dose of at least 36 Gy. Weekly fosaprepitant was well tolerated and improved quality of life for patients without compromising survival.

## Notes

Supplement 1.
Trial Protocol and Statistical Analysis Plan

Supplement 2. **eFigure 1.** Trial Recruitment Flowchart **eFigure 2.** Items of Quality-of-Life Measures With Significant Difference at Different Time Points **eFigure 3.** Kaplan-Meier Curves of Progression-Free Survival **eTable 1.** Baseline Characteristics and Treatment Details **eTable 2.** Dosimetric Parameters **eTable 3.** Pattern of Failure Between Treatment Groups

Supplement 3.
Data Sharing Statement
