# Supplementary material for: Fosaprepitant Weekly vs Every 3 Weeks for the Prevention of Concurrent Chemoradiotherapy–Induced Nausea and Vomiting: A Pilot Randomized Clinical Trial
Source: JAMA Netw Open. 2023 Jul 27;6(7):e2326127. doi: 10.1001/jamanetworkopen.2023.26127 (PMC10375310; doi:10.1001/jamanetworkopen.2023.26127)
Supplement: Supplement 1. — Trial Protocol and Statistical Analysis Plan [file jamanetwopen-e2326127-s001.pdf]

Weekly fosaprepitant for the prevention of nausea and emesis caused by concurrent chemoradiotherapy for nasopharyngeal carcinoma: a pilot randomized clinical trial

Protocol Version

2.0

Clinical correspondence to:

Professor Ming-Yuan Chen

Department of Nasopharyngeal Carcinoma

Sun Yat-sen University Cancer Center

651 Dongfeng Road East

Guangzhou 510060, China

Tel: +86-20-8734-3361

Fax: +86-20-8734-3624

E-mail: chenmy@sysucc.org.cn

Trial profile

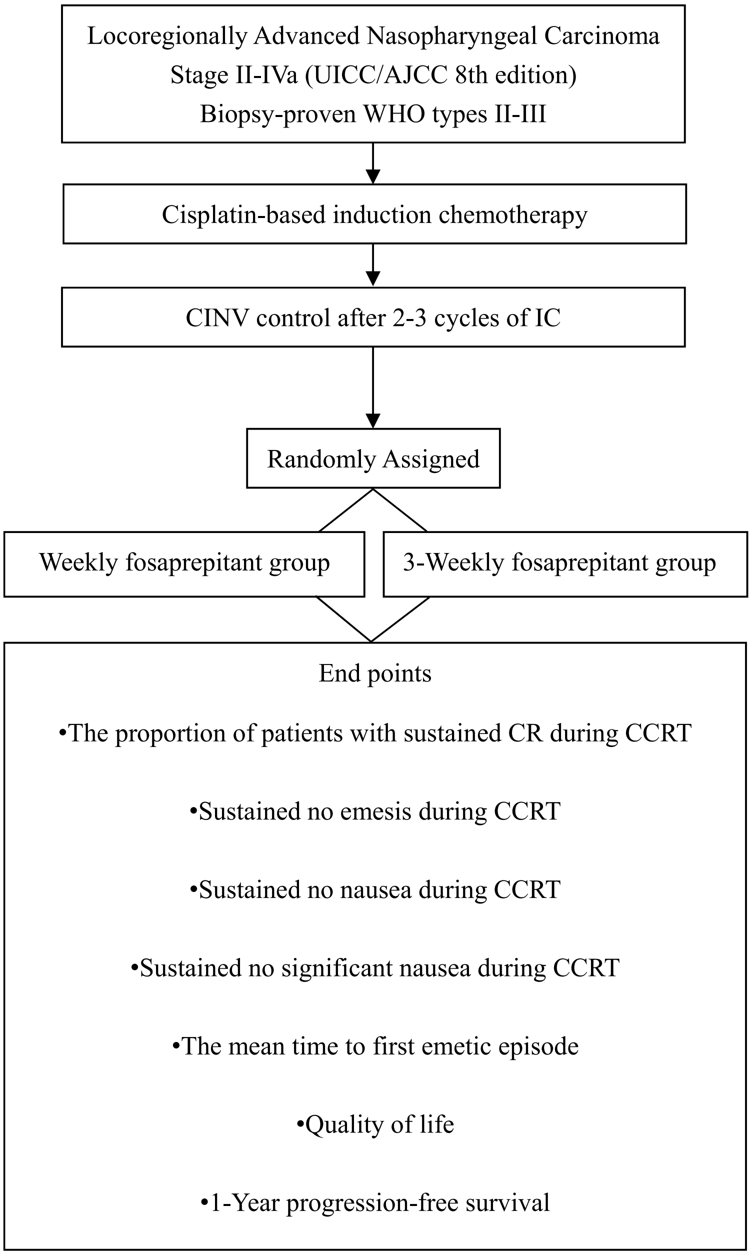

IC, induction chemotherapy; CINV control, defined as no emesis and no use of rescue therapy during the first 120 h after initiation of chemotherapy; CR, complete response (defined as no emesis and no use of rescue therapy); CCRT, concurrent chemoradiotherapy.

1. Background

Nasopharyngeal carcinoma (NPC) is a prevalent disease in China [1]. It has been reported that about 60,000 patients with newly diagnosed NPC are treated in China annually [1]. More than 70% of newly diagnosed NPC present with locoregionally advanced disease [2]. Platinum-based concurrent chemoradiotherapy is the standard treatment for patients with locoregionally advanced nasopharyngeal carcinoma. It has been reported that patients treated with highly emetogenic chemotherapy have a high emetic risk exceeds 90% when not treated with antiemetics [3]. Poorly controlled nausea and vomiting in patients receiving radiotherapy alone or with concomitant chemotherapy that result in substantial impairments in the patient's functional activity and quality of life [4-6]. It can also lengthen the overall radiotherapy time; sometimes patients even refuse further treatment, which can have serious influences on the treatment adherence and survival outcome [7-9]. Unlike substantial evidence in the prevention of chemotherapy-induced nausea and vomiting (CINV), research in the prevention of nausea and vomiting caused by concurrent chemoradiotherapy (CCRT) is currently lacking.

Recent guidelines have classified chemotherapeutic agents into four categories of emesis risk without the use of preventive agents: high (> 90%), moderate (30%-90%), low (10%-30%), and minimal (< 10%) [3, 10]. Currently available antiemetic agents, including corticosteroids, 5-hydroxytryptamine3 (5-HT3) receptor antagonists, and neurokinin-1 receptor antagonists (NK-1RA), are used alone or in combination according to the level of emetogenic potential as prophylaxis against the development of CINV during the acute period (up to 24 hours after chemotherapy) and the delayed period (up to 5 days after treatment) [3, 10-13]. The first-generation 5-hydroxytryptamine 3 receptor antagonist (5-HT3RA) can control acute vomiting by 52.9%-68.8%, but it is not effective in controlling delayed vomiting [14, 15]. The second-generation 5-HT3RA palonosetron can significantly improve the control rate of delayed emesis, but in clinical practice, the incidence of CINV is still as high as 50% [16]. A new class of agents for CINV was introduced with aprepitant, a novel NK-1 antagonist approved in 2003. This agent blocks the neurokinin receptor and thus enhances the activity of 5-HT3 receptor antagonists with a complementary mechanism of action [17, 18]. The triple antiemetic regimen of aprepitant plus a HT3RA and dexamethasone improves the efficacy of CINV compared with the traditional dual antiemetic regimen of 5-HT3RA and dexamethasone [19-22]. Newer agents, including the second-generation 5-HT3RA and the NK-1 antagonist aprepitant, provide additional clinical benefit in

highly and moderately emetogenic therapy. However, aprepitant has the limitations of inconvenient dosing for cancer patients unable to take the medicine orally, different daily doses prone to medication errors, and bioavailability of only 60-65%. Therefore, an equally effective, single-dose alternative to the 3-day regimen for aprepitant that could maintain overall therapeutic benefit with greater convenience and adherence is urgently needed.

Fosaprepitant is the prodrug of the NK-1RA aprepitant and can be rapidly converted to the active form (aprepitant) after intravenous administration [23-25]. The availability of the intravenous route is more feasible in patients with oral mucositis, which is the most common radiation reaction during CCRT and occurs in 83% to 100% of patients with NPC receiving CCRT [26-29]. Data from previous studies show that fosaprepitant is noninferior to aprepitant in the management of CINV [30, 31]. EASE study is a randomized, double-blind, active-control design used to test whether fosaprepitant is noninferior to aprepitant [30]. Patients receiving cisplatin ≥ 70 mg/m2 for the first time received ondansetron and dexamethasone with a aprepitant regimen (125 mg on day 1, 80 mg on day 2, 80 mg on day 3) or a single-dose fosaprepitant regimen (150 mg on day 1). A total of 2,322 patients were randomly assigned, and 2,247 were evaluable for efficacy. The results demonstrated that single-dose intravenous fosaprepitant (150 mg) was noninferior to standard 3-day oral aprepitant in preventing CINV induced by highly emetogenic chemotherapy (HEC) given with ondansetron and dexamethasone. In addition, it has also been reported that the triple antiemetic regimen containing fosaprepitant can prevent CINV caused by moderately emetogenic chemotherapy (MEC) [32, 33]. Considering all these data, fosaprepitant is effective in the management of CINV, particularly for patients receiving moderately and highly emetogenic chemotherapy.

Current guidelines suggest that patients undergoing radiotherapy and concomitant chemotherapy should receive antiemetic prophylaxis according to the guidelines for chemotherapy if the risk level of concomitant chemotherapy is higher than that of radiotherapy [10]. As for the best antiemetic prophylaxis in NPC during CCRT, a combination therapy with a NK-1RA plus a 5-HT3RA and a corticosteroid for the prevention of CINV induced by concomitant high-dose cisplatin should be recommended as the standard treatment, especially when considering that radiation of the head and neck has traditionally been regarded as a low emetic risk treatment [3]. However, the current guideline recommendations mainly come from the pre-intensity-modulated radiation therapy (IMRT) era [7, 10,

34]. Recently, a higher incidence of nausea and vomiting has been reported in patients with head and neck squamous cell carcinomas (HNSCC) treated with IMRT due to a higher dose deposited on the brainstem considering the conformity [6], and nausea and vomiting in NPC during IMRT may be underestimated. The GAND-emesis study has investigated a NK-1RA during radiotherapy and concomitant weekly cisplatin in cervical cancer [4]. The study only included women with cervical cancer and the emetic risk of concomitant low-dose cisplatin (40 mg/m²) is less than high-dose cisplatin. Until now, there has been no evidence regarding the best antiemetic prophylaxis during the entire course of radiotherapy and concomitant HEC.

Against this background, we undertake this randomized prospective pilot study in patients with NPC to compare the efficacy and safety of weekly (once every week) versus 3-weekly (once every 3 weeks) fosaprepitant for the prevention of nausea and emesis caused by IMRT and concomitant HEC among patients with NPC.

2. Objectives of the Study

2.1 To evaluate the efficacy of weekly fosaprepitant for the prevention of nausea and vomiting during radiotherapy and concomitant 3-weekly cisplatin at a dose of 100 mg/m².

2.2 To compare toxicity profile, compliance of the treatment and quality of life between weekly fosaprepitant and 3-weekly fosaprepitant groups.

3. Trial Design

This is an open-label, single center, randomized prospective pilot study to compare the efficacy of weekly versus 3-weekly fosaprepitant regimens for the prevention of nausea and emesis during concurrent chemoradiotherapy for nasopharyngeal carcinoma (NPC) at a single institution. Eligible patients will be randomized to 2 arms at 1:1 ratio.

- Weekly Arm: fosaprepitant 150mg/m^2 weekly in concurrent with radiotherapy during concurrent chemoradiotherapy.
- 3-weekly Arm: fosaprepitant 150mg/m^2 3-weekly in concurrent with chemotherapy during concurrent chemoradiotherapy.

4. Patient Selection and Eligibility Criteria

Inclusion:

• Previously untreated, histologically confirmed nonkeratinizing NPC, including WHO type II or III;

• Stage II - IVa patients (according to the American Joint Committee on Cancer classification system, eighth edition);

• Between 18 and 70 years old;

• Male and no pregnant female;

• Achieved CINV control (defined as no emesis and no use of rescue therapy during the first 120 h after initiation of chemotherapy) using fosaprepitant-based regimens after 2-3 cycles of induction chemotherapy;

• Scheduled to receive IMRT and concomitant 3-weekly cisplatin 100 mg/m²;

• Adequate haematological function: leucocyte count ≥4000/μL, hemoglobin ≥90 g/L and platelet count ≥100000/μL;

• Adequate hepatic function: alanine aminotransferase and aspartate aminotransferase ≤2·0 times the upper limit of normal range;

• Adequate renal function: creatinine clearance ≥60 ml/min;

• Satisfactory performance status: Karnofsky scale (KPS) ≥ 70.

Exclusion:

• Evidence of relapse or distant metastasis;

• Pregnancy or lactation;

• History of prior malignancy or previous treatment for NPC;

• Had other current malignant diagnoses apart from nonmelanoma skin cancers;

• Emesis or clinically significant nausea (moderate or severe) in the 24 h before the first dose of study medication

• Patients who were administered drugs with antiemetic activity within the 24 h before receiving the first dose of study medication;

• Known history of central nervous system disease (e.g., a seizure disorder or brain metastases);

• Any severe intercurrent disease, which may bring unacceptable risk or affect the compliance of the trial, for example, unstable cardiac disease requiring treatment, renal disease, chronic hepatitis, diabetes

with poor control, and emotional disturbance.

5. Randomization, registration, and treatment allocation

5.1 Registration & Randomization

Patients are initially registered to the trial after obtaining informed consent. Randomization procedures will be conducted by sealed envelopes at the Clinical Trials Centre of Sun Yat-sen University Cancer Centre, with a computer-generated random number code.

5.2 Allocation of treatment

All included patients will be randomly assigned in blocks of six (known only to the data management team).

6. Treatment

6.1 Antiemetic therapies

All patients receive a 5-HT3RA (0·25 mg of palonosetron intravenously, 1 mg of granisetron intravenously, or 8 mg of ondansetron intravenously, with the specific agent chosen by the primary clinician) on day 1 and 10 mg of dexamethasone intravenously on days 1 through 4 of concomitant 3-weekly cisplatin. In addition, 150 mg of fosaprepitant is given weekly or 3-weekly as a 20–30-min intravenous infusion from the first dose of cisplatin. Patients are allowed to receive rescue therapy for definite nausea or vomiting.

6.2 Chemotherapy

cisplatin-based induction chemotherapy Cycles were repeated every 3 weeks for at least two cycles. The induction chemotherapy (IC) regimen consist of cisplatin (≥70 mg/m^2) every three week for at least two cycles (a maximum of three cycles). The chemotherapy component of the CCRT regimen consisted of 100 mg/m^2 cisplatin given as a 2 h intravenous infusion every three week for two cycles, beginning on the first day of radiotherapy. In both procedures, cisplatin infusion was preceded by hydration, and mannitol was given concurrently with the cisplatin infusion.

6.3 Radiotherapy

All patients will receive radiotherapy using intensity-modulated radiotherapy (IMRT). Target volumes were defined in accordance with the International Commission on Radiation Units and Measurements (ICRU) reports 50 and 62. The principle of target volume determination for IMRT and prescribed dose

and fractionation are as follows: GTVnx included the sum of the primary tumor volume and the enlarged retropharyngeal nodes, while GTVnd was the volume of clinically involved gross lymph nodes. The high-risk clinical target volume (CTV1) was extended from GTVnx plus a 5–10-mm margin (2–3 mm posteriorly, determined by adjacent brain stem or spinal cord) to encircle the potential high-risk microscopic invaded area. The low-risk clinical target volume (CTV2) was determined on the basis of CTV1 plus a 5–10-mm margin (2–3 mm posteriorly, determined by adjacent brain stem or spinal cord) to encircle the low-risk microscopic invaded area. Planning target volumes (PTVs) were decided by adding a three-dimensional margin of 3–5 mm to the delineated target volume to compensate for the uncertainties in the process of radiotherapy administration. A 3-mm margin was given to spare critical organs including brainstem and spinal cord, to form the planning organ at risk volume (PRV). All patients will be treated with IMRT using simultaneously integrated boost, 5 fractions per week. The prescribed dose was 66–70 Gy, 64–70 Gy, 60–62 Gy, and 54–56 Gy, in 30–33 fractions.

7. Statistical Considerations

7.1 Endpoint Definitions

7.1.1 Primary:

• The proportion of subjects with sustained CR (no emesis and no use of rescue therapy) overall during CCRT.

7.1.2 Secondary:

• The proportion of subjects with sustained no emesis overall during CCRT;

• Sustained no nausea overall during CCRT;

• Sustained no significant nausea (defined as no or mild nausea) overall during CCRT;

• The mean time to first emetic episode;

• Quality of life;

• 1-year progression-free survival (PFS).

7.2 Sample size

This study followed a selection (or pick-the-winner) design, which compares two possible groups for a larger, phase III trial. Prior to the study, data from our previous study showed a CR rate of 40% during CCRT [35]. Based on this value and assuming a 20% difference between groups, a sample size of 36 patients (18 per group) would provide a 85.6% probability of correctly selecting the superior group. A total sample size of 40 was planned to allow for approximately 10% drop-out rate.

7.3 Safety indicators

Acute toxicities are assessed according to NCI-CTC version 5.0. Acute toxicities include hematological toxicity, mucositis, allergic reactions and other adverse events and serious adverse events.

7.4 Quality of Life

EORTC QLQ-C30 and QLQ-H&N35 (V1.0) are used to assess life quality of patients, and the change of their life quality is recorded and evaluated weekly (Week1-7) from before the beginning of treatment to end.

8. Research Governance

8.1 Trial Administration and Logistics

Sun Yat-sen University sponsored this study and was responsible for trial management and audit. Our principal investigator is Professor Ming-Yuan Chen.

8.2 Forms

Patients will complete a study diary throughout the study to record the degree of nausea, the timing and number of emetic episodes, and the use of rescue therapy. Patients estimated the extent of their nausea using a validated four-graded scale (none, mild, moderate, or severe) [17]. Diaries are reviewed by a study nurse to determine the ongoing eligibility. If emesis occurr, the patient will go off the study thereafter. Patients are also asked weekly during treatment to complete two self-administered questionnaires, the European Organization for Research and Treatment of Cancer (EORTC) Quality of Life Questionnaire C30 (QLQ C30) and the EORTC QLQ Head and Neck Cancer–Specific Module (H&N35) [18] to assess the effect of nausea and emesis on their quality of life.

9. Ethical Considerations

The study will be undertaken in accordance with the Declaration of Helsinki. It is the responsibility of the principal investigator to to obtain site-specific approval of the trial protocol from the ethics

committee or institutional review board of each centre. It is the responsibility of the investigator to give each patient full and adequate verbal and written information regarding the objective and procedures of the study and the possible risks involved prior to inclusion in the study. Written informed consents must be given to each patient before enrolment.

10. References

[1] Chen YP, Chan ATC, Le QT, Blanchard P, Sun Y, Ma J. Nasopharyngeal carcinoma. Lancet (London, England). 2019;394:64-80.

[2] Mao YP, Xie FY, Liu LZ, Sun Y, Li L, Tang LL, et al. Re-evaluation of 6th edition of AJCC staging system for nasopharyngeal carcinoma and proposed improvement based on magnetic resonance imaging. International journal of radiation oncology, biology, physics. 2009;73:1326-34.

[3] Hesketh PJ, Kris MG, Basch E, Bohlke K, Barbour SY, Clark-Snow RA, et al. Antiemetics: ASCO Guideline Update. J Clin Oncol. 2020;38:2782-97.

[4] Ruhlmann CH, Christensen TB, Dohn LH, Paludan M, Rønnengart E, Halekoh U, et al. Efficacy and safety of fosaprepitant for the prevention of nausea and emesis during 5 weeks of chemoradiotherapy for cervical cancer (the GAND-emesis study): a multinational, randomised, placebo-controlled, double-blind, phase 3 trial. Lancet Oncol. 2016;17:509-18.

[5] Radiation-induced emesis: a prospective observational multicenter Italian trial. The Italian Group for Antiemetic Research in Radiotherapy. International journal of radiation oncology, biology, physics. 1999;44:619-25.

[6] Maranzano E, De Angelis V, Pergolizzi S, Lupattelli M, Frata P, Spagnesi S, et al. A prospective observational trial on emesis in radiotherapy: analysis of 1020 patients recruited in 45 Italian radiation oncology centres. Radiotherapy and oncology : journal of the European Society for Therapeutic Radiology and Oncology. 2010;94:36-41.

[7] Paiar F, Cristaudo A, Gonnelli A, Giannini N, Cocuzza P, Montrone S, et al. Radiation-induced nausea and vomiting in head and neck cancer: Is it something worth considering in the intensity modulated radiotherapy era? "A narrative review". Head & neck. 2020;42:131-7.

[8] Fowler JF, Lindstrom MJ. Loss of local control with prolongation in radiotherapy. International journal of radiation oncology, biology, physics. 1992;23:457-67.

[9] Robertson C, Robertson AG, Hendry JH, Roberts SA, Slevin NJ, Duncan WB, et al. Similar

decreases in local tumor control are calculated for treatment protraction and for interruptions in the radiotherapy of carcinoma of the larynx in four centers. International journal of radiation oncology, biology, physics. 1998;40:319-29.

[10] Ruhlmann CH, Jahn F, Jordan K, Dennis K, Maranzano E, Molassiotis A, et al. 2016 updated MASCC/ESMO consensus recommendations: prevention of radiotherapy-induced nausea and vomiting. Support Care Cancer. 2017;25:309-16.

[11] Rao KV, Faso A. Chemotherapy-induced nausea and vomiting: optimizing prevention and management. Am Health Drug Benefits. 2012;5:232-40.

[12] Hesketh PJ, Kris MG, Grunberg SM, Beck T, Hainsworth JD, Harker G, et al. Proposal for classifying the acute emetogenicity of cancer chemotherapy. J Clin Oncol. 1997;15:103-9.

[13] Kris MG, Gralla RJ, Clark RA, Tyson LB, O'Connell JP, Wertheim MS, et al. Incidence, course, and severity of delayed nausea and vomiting following the administration of high-dose cisplatin. J Clin Oncol. 1985;3:1379-84.

[14] Hickok JT, Roscoe JA, Morrow GR, Bole CW, Zhao H, Hoelzer KL, et al. 5-Hydroxytryptamine-receptor antagonists versus prochlorperazine for control of delayed nausea caused by doxorubicin: a URCC CCOP randomised controlled trial. Lancet Oncol. 2005;6:765-72.

[15] Geling O, Eichler HG. Should 5-hydroxytryptamine-3 receptor antagonists be administered beyond 24 hours after chemotherapy to prevent delayed emesis? Systematic re-evaluation of clinical evidence and drug cost implications. J Clin Oncol. 2005;23:1289-94.

[16] Navari RM. Management of chemotherapy-induced nausea and vomiting : focus on newer agents and new uses for older agents. Drugs. 2013;73:249-62.

[17] Navari RM. Role of neurokinin-1 receptor antagonists in chemotherapy-induced emesis: summary of clinical trials. Cancer Invest. 2004;22:569-76.

[18] Massaro AM, Lenz KL. Aprepitant: a novel antiemetic for chemotherapy-induced nausea and vomiting. Ann Pharmacother. 2005;39:77-85.

[19] Hesketh PJ, Grunberg SM, Gralla RJ, Warr DG, Roila F, de Wit R, et al. The oral neurokinin-1 antagonist aprepitant for the prevention of chemotherapy-induced nausea and vomiting: a multinational, randomized, double-blind, placebo-controlled trial in patients receiving high-dose cisplatin--the Aprepitant Protocol 052 Study Group. J Clin Oncol. 2003;21:4112-9.

[20] Poli-Bigelli S, Rodrigues-Pereira J, Carides AD, Julie Ma G, Eldridge K, Hipple A, et al. Addition of the neurokinin 1 receptor antagonist aprepitant to standard antiemetic therapy improves control of chemotherapy-induced nausea and vomiting. Results from a randomized, double-blind, placebo-controlled trial in Latin America. Cancer. 2003;97:3090-8.

[21] Schmoll HJ, Aapro MS, Poli-Bigelli S, Kim HK, Park K, Jordan K, et al. Comparison of an aprepitant regimen with a multiple-day ondansetron regimen, both with dexamethasone, for antiemetic efficacy in high-dose cisplatin treatment. Ann Oncol. 2006;17:1000-6.

[22] Roila F, Ruggeri B, Ballatori E, Del Favero A, Tonato M. Aprepitant versus dexamethasone for preventing chemotherapy-induced delayed emesis in patients with breast cancer: a randomized double-blind study. J Clin Oncol. 2014;32:101-6.

[23] Hale JJ, Mills SG, MacCoss M, Dorn CP, Finke PE, Budhu RJ, et al. Phosphorylated morpholine acetal human neurokinin-1 receptor antagonists as water-soluble prodrugs. Journal of medicinal chemistry. 2000;43:1234-41.

[24] Huskey SE, Luffer-Atlas D, Dean BJ, McGowan EM, Feeney WP, Chiu SH. Substance P receptor antagonist I: conversion of phosphoramidate prodrug after i.v. administration to rats and dogs. Drug metabolism and disposition: the biological fate of chemicals. 1999;27:1367-73.

[25] Tattersall FD, Rycroft W, Cumberbatch M, Mason G, Tye S, Williamson DJ, et al. The novel NK1 receptor antagonist MK-0869 (L-754,030) and its water soluble phosphoryl prodrug, L-758,298, inhibit acute and delayed cisplatin-induced emesis in ferrets. Neuropharmacology. 2000;39:652-63.

[26] Zheng B, Zhu X, Liu M, Yang Z, Yang L, Lang J, et al. Randomized, Double-Blind, Placebo-Controlled Trial of Shuanghua Baihe Tablets to Prevent Oral Mucositis in Patients With Nasopharyngeal Cancer Undergoing Chemoradiation Therapy. International journal of radiation oncology, biology, physics. 2018;100:418-26.

[27] Peng G, Wang T, Yang KY, Zhang S, Zhang T, Li Q, et al. A prospective, randomized study comparing outcomes and toxicities of intensity-modulated radiotherapy vs. conventional two-dimensional radiotherapy for the treatment of nasopharyngeal carcinoma. Radiotherapy and oncology : journal of the European Society for Therapeutic Radiology and Oncology. 2012;104:286-93.

[28] Yang G, Feng D, Li F, Luo B, Zhu J, Yang Q, et al. A randomized, controlled phase II trial of maxillofacial and oral massage in attenuating severe radiotherapy-induced oral mucositis and lipid

metabolite changes in nasopharyngeal carcinoma. Radiotherapy and oncology : journal of the European Society for Therapeutic Radiology and Oncology. 2021;163:76-82.

[29] Hua X, Chen LM, Zhu Q, Hu W, Lin C, Long ZQ, et al. Efficacy of controlled-release oxycodone for reducing pain due to oral mucositis in nasopharyngeal carcinoma patients treated with concurrent chemoradiotherapy: a prospective clinical trial. Supportive care in cancer : official journal of the Multinational Association of Supportive Care in Cancer. 2019;27:3759-67.

[30] Grunberg S, Chua D, Maru A, Dinis J, DeVandry S, Boice JA, et al. Single-dose fosaprepitant for the prevention of chemotherapy-induced nausea and vomiting associated with cisplatin therapy: randomized, double-blind study protocol--EASE. J Clin Oncol. 2011;29:1495-501.

[31] Saito H, Yoshizawa H, Yoshimori K, Katakami N, Katsumata N, Kawahara M, et al. Efficacy and safety of single-dose fosaprepitant in the prevention of chemotherapy-induced nausea and vomiting in patients receiving high-dose cisplatin: a multicentre, randomised, double-blind, placebo-controlled phase 3 trial. Ann Oncol. 2013;24:1067-73.

[32] Weinstein C, Jordan K, Green SA, Camacho E, Khanani S, Beckford-Brathwaite E, et al. Single-dose fosaprepitant for the prevention of chemotherapy-induced nausea and vomiting associated with moderately emetogenic chemotherapy: results of a randomized, double-blind phase III trial. Ann Oncol. 2016;27:172-8.

[33] Nishimura J, Satoh T, Fukunaga M, Takemoto H, Nakata K, Ide Y, et al. Combination antiemetic therapy with aprepitant/fosaprepitant in patients with colorectal cancer receiving oxaliplatin-based chemotherapy (SENRI trial): a multicentre, randomised, controlled phase 3 trial. Eur J Cancer. 2015;51:1274-82.

[34] Hesketh PJ, Kris MG, Basch E, Bohlke K, Barbour SY, Clark-Snow RA, et al. Antiemetics: American Society of Clinical Oncology Clinical Practice Guideline Update. Journal of clinical oncology : official journal of the American Society of Clinical Oncology. 2017;35:3240-61.

[35] Cao SM, Yang Q, Guo L, Mai HQ, Mo HY, Cao KJ, et al. Neoadjuvant chemotherapy followed by concurrent chemoradiotherapy versus concurrent chemoradiotherapy alone in locoregionally advanced nasopharyngeal carcinoma: A phase III multicentre randomised controlled trial. Eur J Cancer. 2017;75:14-23.
