# Supplementary material for: Fosaprepitant Weekly vs Every 3 Weeks for the Prevention of Concurrent Chemoradiotherapy–Induced Nausea and Vomiting: A Pilot Randomized Clinical Trial
Source: JAMA Netw Open. 2023 Jul 27;6(7):e2326127. doi: 10.1001/jamanetworkopen.2023.26127 (PMC10375310; doi:10.1001/jamanetworkopen.2023.26127)
Supplement: Supplement 2. — eFigure 1. Trial Recruitment Flowchart eFigure 2. Items of Quality-of-Life Measures With Significant Difference at Different Time Points eFigure 3. Kaplan-Meier Curves of Progression-Free Survival eTable 1. Baseline Characteristics and Treatment Details eTable 2. Dosimetric Parameters eTable 3. Pattern of Failure Between Treatment Groups [file jamanetwopen-e2326127-s002.pdf]

Supplemental Online Content

Yang Q, Zou X, Xie YL, et al. Fosaprepitant weekly vs every 3 weeks for the prevention of concurrent chemoradiotherapy–induced nausea and vomiting: a pilot randomized clinical trial. *JAMA Netw Open*. 2023;6(7):e2326127. doi:10.1001/jamanetworkopen.2023.26127

eFigure 1. Trial Recruitment Flowchart
eFigure 2. Items of Quality-of-Life Measures With Significant Difference at Different Time Points
eFigure 3. Kaplan-Meier Curves of Progression-Free Survival
eTable 1. Baseline Characteristics and Treatment Details
eTable 2. Dosimetric Parameters
eTable 3. Pattern of Failure Between Treatment Groups

This supplemental material has been provided by the authors to give readers additional information about their work.

© 2023 Yang Q et al. *JAMA Network Open.*

eFigure 1. Trial Recruitment Flowchart

IC, induction chemotherapy; CINV, chemotherapy-induced nausea and vomiting; ITT, intention-to-treat; CCRT, concurrent chemoradiotherapy.

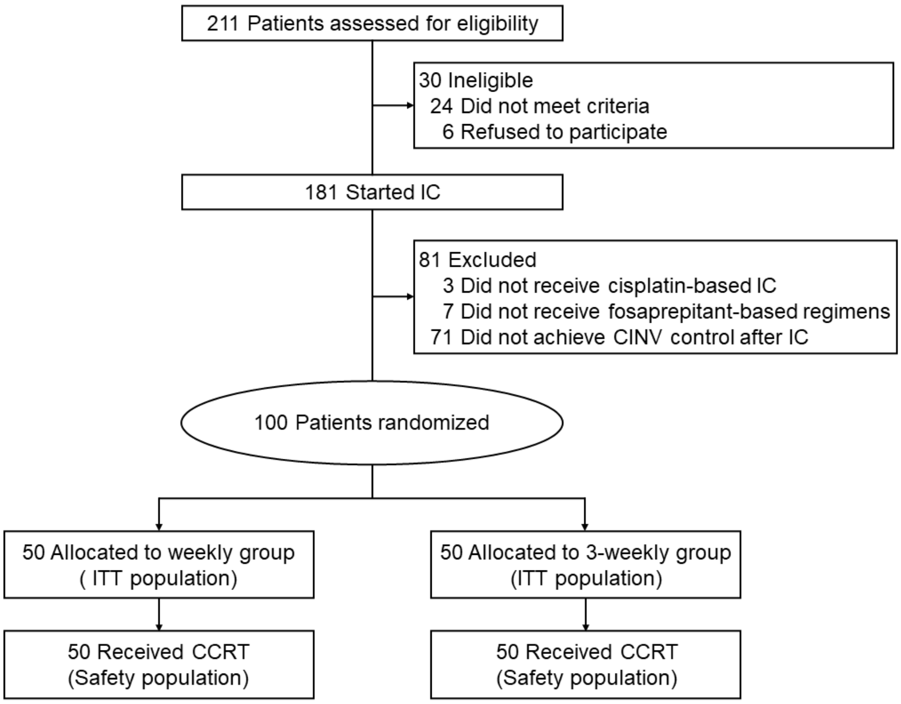

© 2023 Yang Q et al. *JAMA Network Open.*

eFigure 2. Items of Quality -of-Life Measures With Significant Difference at Different Time Points

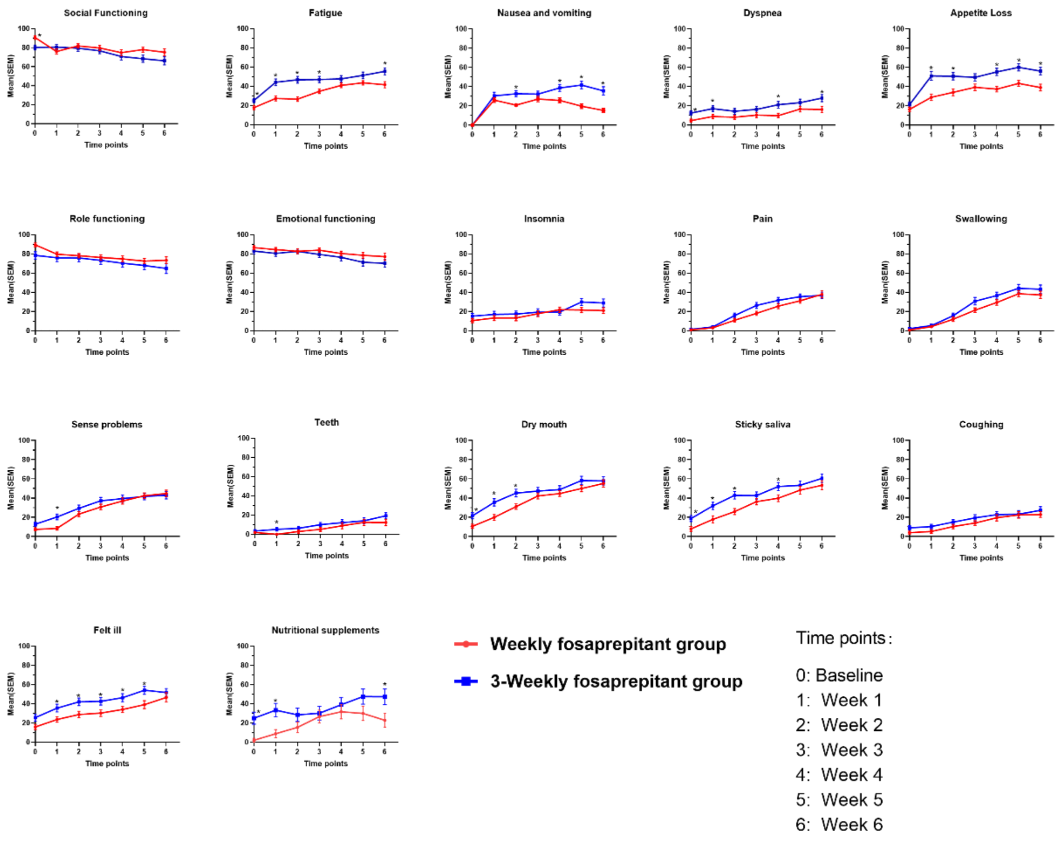

© 2023 Yang Q et al. *JAMA Network Open.*

eFigure 3. Kaplan-Meier Curves of Progression-Free Survival

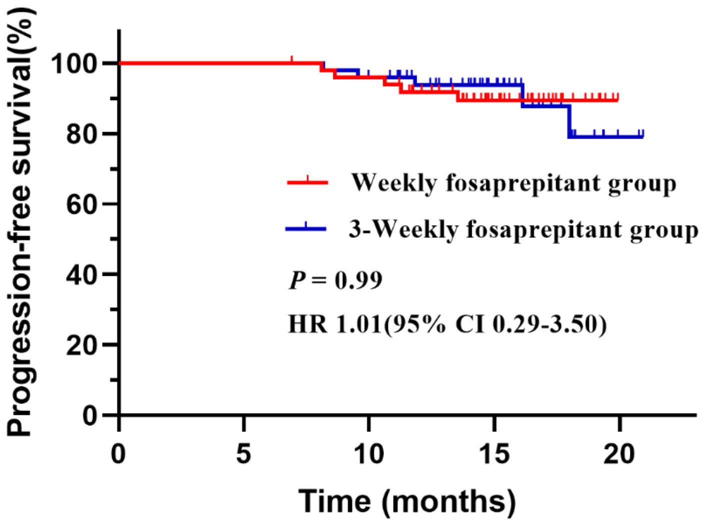

Number at risk					
Weekly fosaprepitant group	50	50	48	25	1
3-Weekly fosaprepitant group	50	50	48	26	3

© 2023 Yang Q et al. *JAMA Network Open.*

eTable 1. Baseline Characteristics and Treatment Details

Characteristic, No. (%)	Weekly group (n=50)	Triweekly group (n=50)
Age, median (IQR)	43 (36-54)	49.5 (41-59)
Sex		
Male	41 (82)	42 (84)
Female	9 (18)	8 (16)
T category		
T2	6 (12)	5 (10)
T3	28 (56)	31 (62)
T4	16 (32)	14 (28)
N category		
N0	2 (4)	2 (4)
N1	19 (38)	18 (36)
N2	17 (34)	23 (46)
N3	12 (24)	7 (14)
Stage		
II	3 (6)	2 (4)
III	24 (48)	28 (56)
IVa	23 (46)	20 (40)
Induction chemotherapeutic agent		
GP	40 (80)	42 (84)
PF	9 (18)	5 (10)
TPF	1 (2)	3 (6)
Induction chemotherapeutic cycle		
Two	2 (4)	3 (6)
Three	48 (96)	47 (94)

Data are n (%), or median (range). IQR=interquartile range; GP=cisplatin and gemcitabine; PF=cisplatin and fluorouracil; TPF=cisplatin, fluorouracil, and paclitaxel.

© 2023 Yang Q et al. *JAMA Network Open.*

eTable 2. Dosimetric Parameters

Characteristic, Median(Range)	Weekly group (n=50)	Triweekly group (n=50)	*P*
GTV volume, cm^3	61.75(21.7-205.0)	55.85 (24.0-187.2)	0.29
CTV1 volume, cm^3	147.75(63.6-307.0)	140.05(67.4-327.5)	0.15
CTV2 volume, cm^3	593.00(356.4-1350.6)	562.25(375.7-997.5)	0.30
Brain stem			
Dmin, cGy	884.40(328.2-2214.8)	699.55(251.4-2536.3)	0.14
Dmax, cGy	5831.20(4243.2-6575.9)	5549.60(3556.9-6768.8)	0.21
Dmean, cGy	3309.95(2149.5-4368.8)	3002.25(1713.0-4246.8)	0.20
Dmedian, cGy	3549.75(2151.8-4446.9)	3148.75(1627.9-4324.6)	0.20
Vestibules (left)			
Dmin, cGy	3667.60(2281.5-6796.5)	3211.65(2196.7-5076.3)	0.26
Dmax, cGy	4853.55(2396.8-7330.8)	4304.40(2654.8-6783.2)	0.17
Dmean, cGy	4126.65(2603.6-7208.5)	3686.45(2452.2-5921.5)	0.20
Dmedian, cGy	4104.45(2600.5-7228.3)	3677.70(2445.7-5922.6)	0.21
Vestibules (right)			
Dmin, cGy	3633.25(2102.2-5039.5)	3608.25(1875.4-5383.8)	0.64
Dmax, cGy	4733.35(2652.9-6852.7)	4728.25(2666.8-6676.7)	0.83
Dmean, cGy	4146.00(2372.4-5862.8)	4143.95(2302.0-6005.7)	0.66
Dmedian, cGy	4130.95(2371.6-5862.2)	4116.5(2308.0-6030.1)	0.64

NOTE. *P* value is derived from the Mann-Whitney U test in continuous variable.

Abbreviations: Dmin = minimum dose; Dmax = maximum dose; Dmean = mean dose; Dmedian = median dose.

© 2023 Yang Q et al. *JAMA Network Open.*

eTable 3. Pattern of Failure Between Treatment Groups

Events, No. (%)	Weekly group (n=50)	Triweekly group (n=50)	All (n=100)
Progression	5 (10)	5 (10)	10 (10)
Distant metastasis	4 (8)	4 (8)	8 (8)
Local relapse	1 (2)	1 (2)	2 (8)
Nodal relapse	0	0	0
Death	0	0	0

© 2023 Yang Q et al. *JAMA Network Open.*
