# Supplementary material for: Fosaprepitant Weekly vs Every 3 Weeks for the Prevention of Concurrent Chemoradiotherapy–Induced Nausea and Vomiting: A Pilot Randomized Clinical Trial
Source: JAMA Netw Open. 2023 Jul 27;6(7):e2326127. doi: 10.1001/jamanetworkopen.2023.26127 (PMC10375310; doi:10.1001/jamanetworkopen.2023.26127)
Supplement: Supplement 3. — Data Sharing Statement [file jamanetwopen-e2326127-s003.pdf]

Data Sharing Statement

Yang. Fosaprepitant Weekly vs Every 3 Weeks for the Prevention of Concurrent Chemoradiotherapy–Induced Nausea and Vomiting. *JAMA Netw Open*. Published July 27, 2023. doi:10.1001/jamanetworkopen.2023.26127

Data
Data available: Yes
Data types: Data (not involving human participants)
How to access data: yangqi@sysucc.org.cn
When available: With publication

Supporting Documents
Document types: None

Additional Information
Who can access the data: yangqi@sysucc.org.cn
Types of analyses: for any purpose
Mechanisms of data availability: after approval of a proposal
